# P4HB promotes HCC tumorigenesis through downregulation of GRP78 and subsequent upregulation of epithelial-to-mesenchymal transition

**DOI:** 10.18632/oncotarget.14337

**Published:** 2016-12-28

**Authors:** Wei Xia, Juhua Zhuang, Guoyu Wang, Jing Ni, Jiening Wang, Ying Ye

**Affiliations:** ^1^ Department of Nuclear Medicine, Seventh People's Hospital of Shanghai University of TCM, Shanghai, China; ^2^ Department of Integrated TCM & western medcine, President's Office of Seventh People's Hospital of Shanghai University of TCM, Shanghai, China

**Keywords:** P4HB, GRP78, hepatocellular carcinoma, tumorigenesis, epithelial-to-mesenchymal transition

## Abstract

P4HB and GRP78 are molecular chaperones involved in cellular response to ER stress. They have been linked to cancer progression; however, their roles in hepatocellular carcinoma (HCC) are largely unclear. In this study, we found that P4HB is overexpressed in human HCC tissues and cell lines. Higher tumoral P4HB levels are correlated with more advanced disease and poorer survival. GRP78 expression is inversely correlated with P4HB in human HCC tissues, and downregulated by P4HB in HCC cell lines. P4HB overexpression promotes HCC cell growth, migration, invasion and epithelial-to-mesenchymal transition (EMT) *in vitro*. GRP78 overexpression not only inhibits HCC cell growth, migration, invasion and EMT, but also antagonizes the oncogenic effects of P4HB overexpression. Furthermore, P4HB silencing inhibits HCC tumorigenesis *in vivo*. Taken together, our results provided evidence that P4HB promotes HCC progression through downregulation of GRP78 and subsequent upregulation of EMT.

## INTRODUCTION

Tumor cells are often exposed to hypoxia and nutrient deprivation. These unfavorable conditions result in accumulation of unfolded proteins in the ER, causing ER stress. This triggers a defensive process known as the “unfolded protein response” or UPR. UPR allows tumor cells to adapt to ER stress and thereby safeguards their survival [1]. Compelling evidence shows that UPR is activated in many cancers, and plays a critical role in cancer progression [2–4].

The 78-kilodalton glucose regulated protein (GRP78/BiP) is a multifunctional protein belonging to the heat shock protein 70 family. GRP78 is the most abundant ER chaperone and functions as a gatekeeper of the mammalian UPR [5]. GRP78 promotes tumor growth, survival and metastasis; and is generally considered to play an oncogenic role in cancer [6]. However, a number of studies suggest that GRP78 can also function as a tumor suppressor in certain cancer types. For example, colon cancer cells with surface expression of GRP78 exhibit reduced proliferation, tumor growth, and metastasis, while GRP78 knockdown restores tumorigenicity of these cells [7]. Additionally, GRP78 knockdown increases the migratory ability of colon cancer and hepatocellular carcinoma (HCC) cells through upregulation of epithelial-to-mesenchymal transition (EMT) [8, 9]. These results suggest that the role of GRP78 in tumorigenesis can be context-dependent.

Prolyl 4-hydroxylase, beta polypeptide (P4HB), the beta subunit of prolyl 4-hydroxylase, can act as a ER chaperone to inhibit aggregation of misfolded proteins [10]. Recent studies have shown that P4HB is upregulated in many cancer cell types, and its expression correlates with cancer progression and clinical outcome [11]. For example, higher P4HB levels are associated with more advanced disease and drug resistance in malignant glioma, and P4HB inhibition increases the chemosensitivity of drug-resistant glioma cells [12, 13]. P4HB is overexpressed in non-small cell lung cancer (NSCLC), and decreased P4HB expression following drug treatment predicts better clinical outcome [14]. These findings demonstrate the potential oncogenic properties of P4HB in cancer. However, the role of P4HB in HCC remains unclear.

In this study, we determined P4HB levels in tumor and adjacent normal tissues of HCC patients. We also assessed the correlations between tumoral P4HB and disease stage, metastasis and patient survival. Moreover, the role of P4HB in HCC tumorigenesis *in vitro* and *in vivo*, as well as the underlying mechanisms involving GRP78 were investigated. Our data supported P4HB as a potential diagnostic/prognostic marker and therapeutic target for HCC.

## RESULTS

### P4HB is upregulated in HCC and is inversely correlated with HCC patient survival

**Figure 1:**
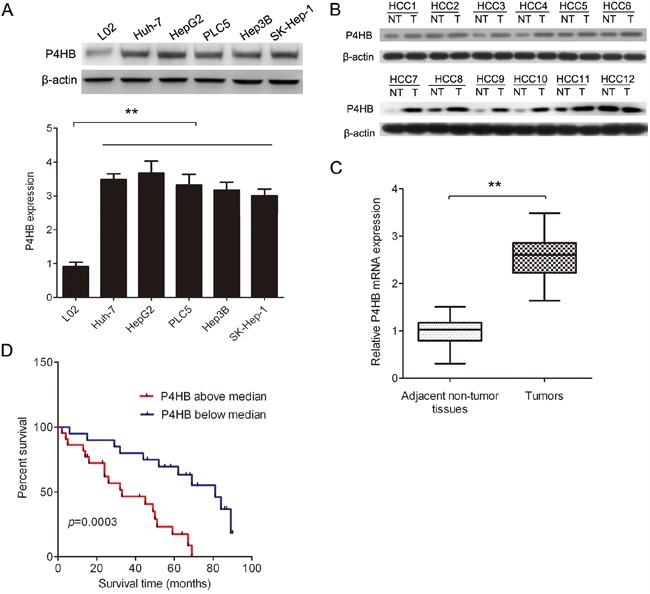
P4HB is upregulated in human HCC and is inversely correlated with HCC patient survival **A.** P4HB protein expression in HCC cell lines (Huh-7, HepG2, PLC5, Hep3B, and SK-Hep-1) and the normal liver cell line L02 by western blotting. β-actin was used as a loading control. n = 3, **P < 0.01. **B.** P4HB protein expression in paired human HCC and adjacent normal liver tissues by western blotting (12 pairs are shown). β-actin was used as a loading control. T, tumor tissue; NT, adjacent normal liver tissue. **C.** P4HB mRNA expression in paired human HCC and adjacent normal liver tissues by qRT-PCR. n = 42, **P<0.01. **D.** Kaplan-Meier analysis of HCC patient survival rate in relation to tumoral P4HB protein expression. P4HB above median, tumoral P4HB > 2.5-fold of adjacent normal tissue; P4HB below median, tumoral P4HB < 2.5-fold of adjacent normal tissue. Patient survival was inversely correlated with tumoral P4HB (P= 0.0003).

To find out whether P4HB is dyregulated in HCC, we determined P4HB mRNA and protein levels in tumor and adjacent normal liver tissues of HCC patients by qRT-PCR and western blotting, respectively. Our data revealed higher P4HB mRNA and protein levels in HCC tissues compared with adjacent normal tissues (Figure 1B, 1C). Moreover, Kaplan-Meier analysis revealed a significant inverse correlation between HCC patient survival rate and tumoral P4HB protein level (Figure 1D, P = 0.0003). These data suggested that P4HB may serve as a diagnostic/prognostic marker for HCC. In addition, the HCC cell lines Huh-7, HepG2, PLC5, Hep3B, and SK-Hep-1 showed significantly higher P4HB protein expression than the normal liver cell line L02 (Figure 1A).

### Higher P4HB levels are correlated with more advanced HCC and metastasis

We subsequently analyzed the correlation between clinicopathological parameters of HCC patients and tumoral P4HB protein levels. We found that P4HB levels were significantly correlated with the grade and stage of the disease, number of tumors, and vascular invasion (Table 1). These findings further supported the prognostic value of P4HB in HCC. However, P4HB levels were not affected by patient gender or age.

**Table 1:** Correlative Analysis of P4HB protein Levels With Clinicopathological Features

ClinicopatholigicParameters	No. of Specimens	P4HB Expression(Tumor/Nontumoral)		P Value
		Low	High	
Sex				NS
Female	35	16	19	
Male	7	4	3	
Age	42	51.6±10.5	53.9±9.8	NS
Grade				NS
1	10	9	1	
2	17	7	10	
3	15	4	11	
Stage				0.009
1	16	12	4	
2 or 3	26	8	18	
Multiple tumor				0.02
No	26	14	12	
Yes	16	6	10	
Vascular invasion (macro)				0.006
No	18	12	6	
Yes	24	8	16	
Vascular invasion (micro)				0.001
No	16	13	3	
Yes	26	7	19	
HBV				NS
No	5	3	2	
Yes	37	17	20	
Cirrhosis				NS
No	22	12	10	
Yes	20	8	12	

High tumoral P4HB expression was regarded at >2.5-fold up-regulation, relative to the adjacent nontumoral liver.

Abbreviation: NS, no significant difference.

### P4HB promotes HCC cell growth, migration, and invasion

**Figure 2:**
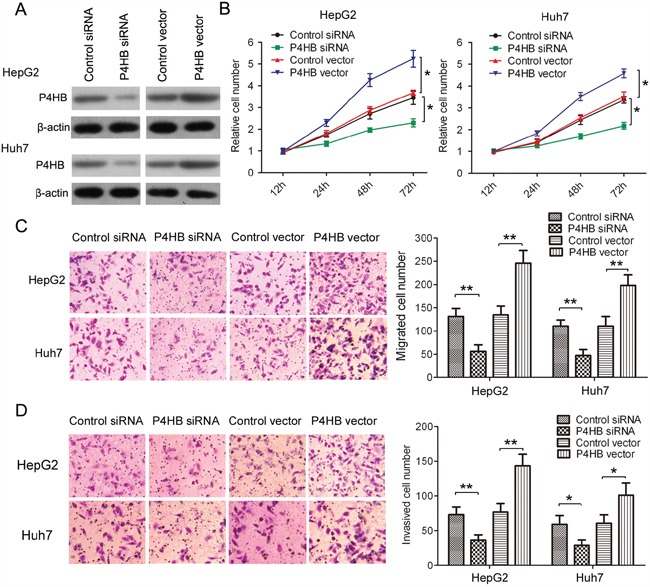
P4HB promotes HCC cell growth, migration, and invasion HepG2 and Huh-7cells were transfected with P4HB siRNA, control siRNA, P4HB, or vector alone for 24 h. **A.** P4HB protein expression determined by western blotting. β-actin was used as a loading control. **B.** Cell proliferation determined by the MTT assay. **C** and **D.** Cell migration (C) and invasion (D) determined by Transwell assays using un-coated and Matrigel-coated membranes, respectively. n = 3, *P<0.05, **P<0.01.

To find out whether P4HB promotes HCC tumorigenesis, we used HepG2 and Huh-7 cells transfected with P4HB or P4HB siRNA. Compared with controls, transfection with P4HB and P4HB siRNA led to enhanced and suppressed P4HB expression, respectively (Figure 2A). Our data showed that P4HB overexpression significantly enhanced HepG2 and Huh-7 cell growth, migration, and invasion while P4HB silencing had the opposite effects (Figure 2B–2D). These results suggested that P4HB plays an oncogenic role in HCC.

### P4HB induces EMT of HCC cells

**Figure 3:**
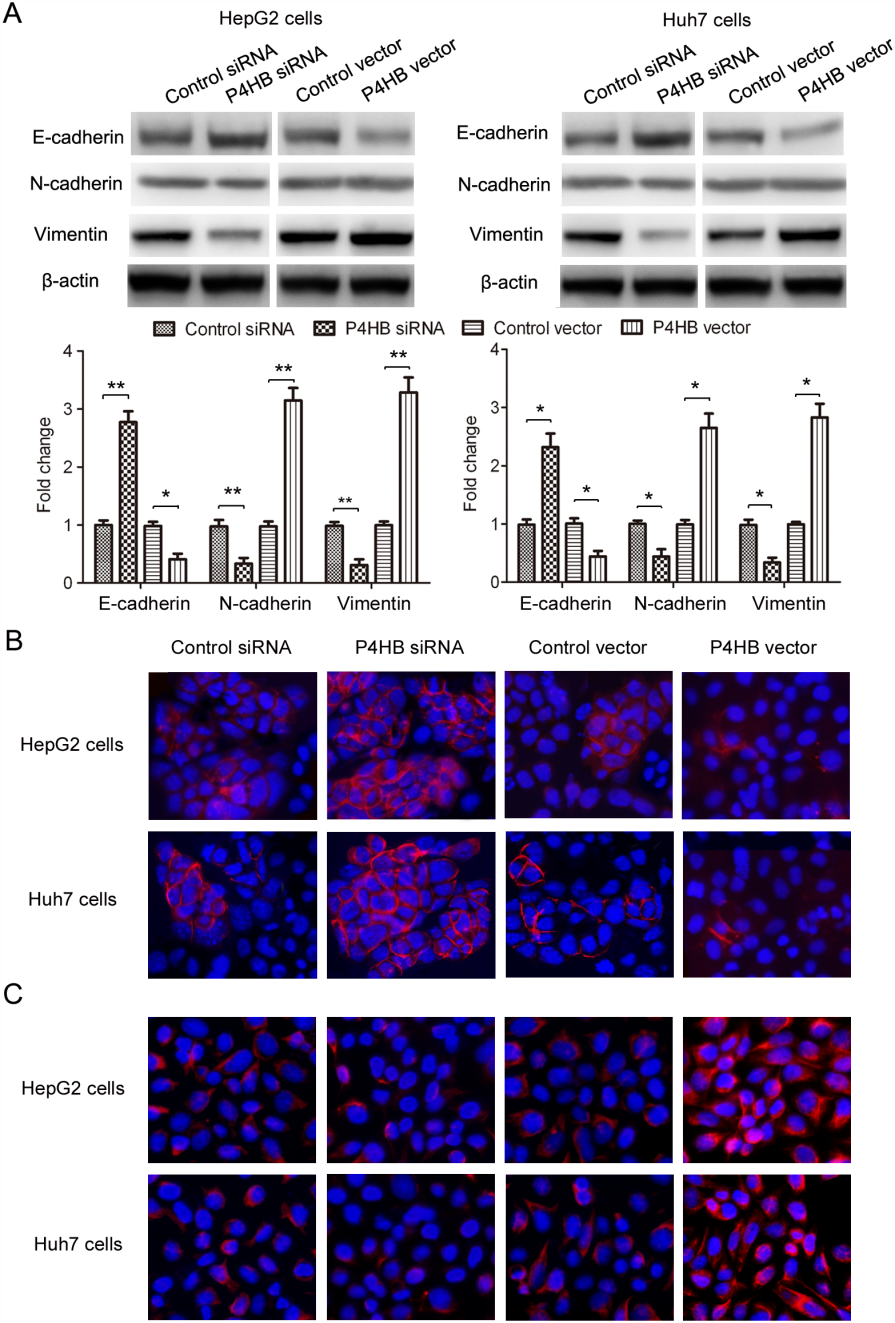
P4HB induces EMT of HCC cells HepG2 and Huh-7 cells were transfected with P4HB siRNA, control siRNA, P4HB, or vector alone for 24 h. **A.** Protein expression of the epithelial marker E-cadherin and the mesenchymal markers N-cadherin and vimentin by western blotting. β-actin was used as a loading control. n = 3, *P<0.05, **P<0.01. **B** and **C.** Immunofluorescence analysis for E-cadherin (B) and vimentin (C) expression.

Epithelial-mesenchymal transition (EMT) plays a key role in tumor progression, invasion, and metastasis [15]. In this study, we examined the function of P4HB in EMT of HCC cells by measuring the epithelial marker E-cadherin and the mesenchymal markers N-cadherin and vimentin. P4HB overexpression in HepG2 and Huh-7 cells led to significantly reduced E-cadherin expression along with increased N-cadherin and vimentin expression; and P4HB silencing showed the opposite effects (Figure 3A). Immunofluorescence staining of E-cadherin and vimentin showed similar results (Figure 3B, 3C). Together, these data indicated that P4HB induces EMT of HCC cells.

### P4HB downregulates GRP78 in HCC

**Figure 4:**
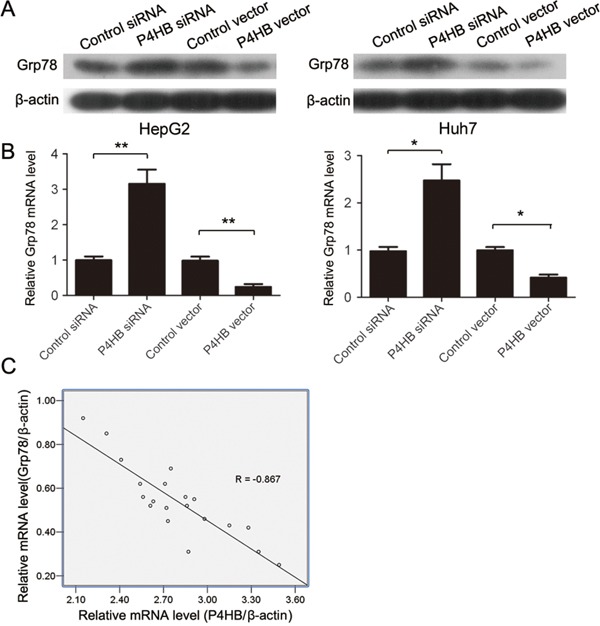
P4HB downregulates GRP78 in human HCC **A, B.** HepG2 and Huh-7 cells were transfected with P4HB siRNA, control siRNA, P4HB, or vector alone for 24 h. GRP78 protein and mRNA expression were determined by western blotting (A) and qRT-PCR (B), respectively. Data were normalized to β-actin. n = 3, *P<0.05, **P<0.01. **C.** Correlation analysis of P4HB and GRP78 mRNA levels in human HCC tissues. Data were normalized to β-actin. n = 20, R = -0.867, P<0.001.

GRP78 has been reported to inhibit EMT of HCC cells [9]. To find out whether GRP78 is involved in EMT of HCC cells induced by P4HB, we examined the possible regulatory relationship between P4HB and GRP78. P4HB overexpression significantly reduced while P4HB silencing significantly increased GRP78 mRNA and protein expression in HepG2 and Huh-7 cells (Figure 4A, 4B). These data suggested that P4HB downregulates GRP78 in human HCC. Indeed, GRP78 mRNA levels in human HCC tissues were found to be inversely correlated with P4HB mRNA levels (Figure 4C).

### P4HB promotes HCC cell EMT, migration, and invasion by downregulating GRP78

**Figure 5:**
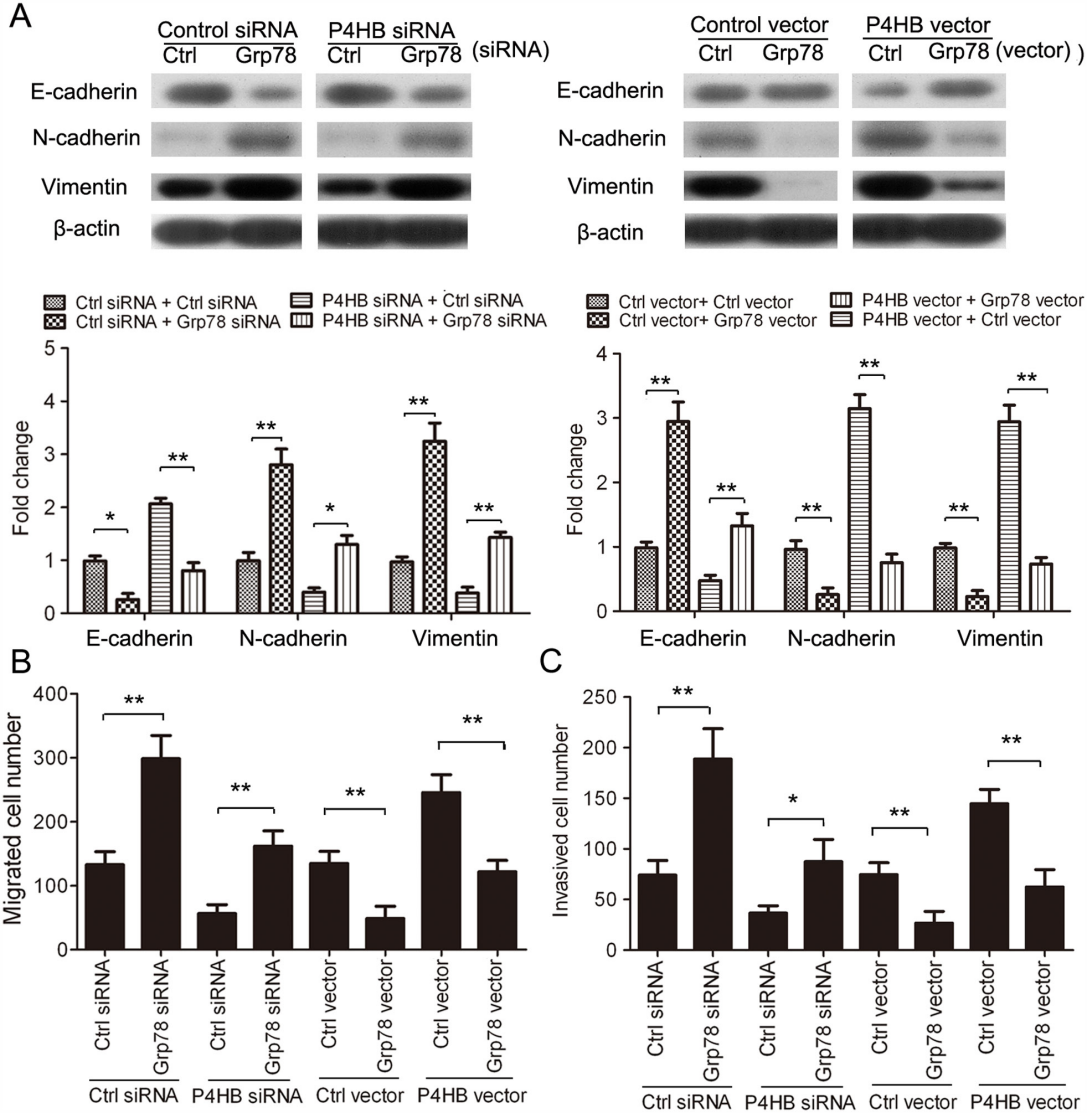
P4HB promotes HCC cell EMT, migration, and invasion by downregulating GRP78 HepG2cells were transfected as indicated for 72 h. **A.** E-cadherin, N-cadherin, and vimentin protein expression by western blotting. β-actin was used as a loading control. **B** and **C.** Cell migration (B) and invasion (C) by Transwell assays using un-coated and Matrigel-coated membranes, respectively. n = 3, *P<0.05, **P<0.01.

Having found that P4HB downregulates GRP78 in HCC, we speculated that the oncogenic properties of P4HB in HCC cells might be mediated by GRP78. To test this hypothesis, we examined the function of GRP78 in HCC cell EMT, migration, and invasion. GRP78 overexpression in HepG2 cells significantly increased E-cadherin expression and decreased N-cadherin and vimentin expression; and GRP78 silencing showed the opposite effects (Figure 5A). Thus, in contrast to P4HB, GRP78 suppresses EMT of HCC cells. Consistent with these results, GRP78 overexpression significantly inhibited HepG2 cell migration and invasion; and GRP78 silencing showed opposite effects (Figure 5B, 5C). Importantly, the effects of P4HB overexpression and silencing on HepG2 EMT, migration, and invasion were reversed by GRP78 overexpression and silencing, respectively (Figure 5A–5C). These findings provided strong evidence that P4HB promotes HepG2 EMT, migration, and invasion by downregulating GRP78.

### P4HB silencing inhibits HCC tumorigenesis *in vivo*

**Figure 6:**
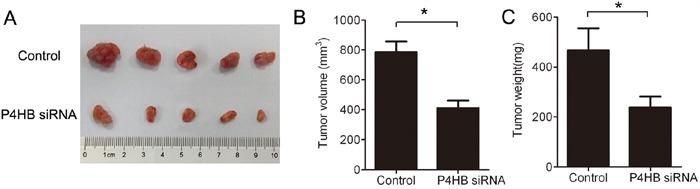
P4HB silencing inhibits HCC tumorigenesis in mice BALB/c nude mice (n = 5 per group) were subcutaneously inoculated with HepG2 cells stably expressing P4HB siRNA or control siRNA. The tumors were harvested on day 37. **A.** Tumor image. **B.** Tumor volume. **C.** Tumor weights. *P<0.05.

To find out whether our *in vitro* data can be extrapolated to *in vivo* situation, we established a mouse xenograft model of human HCC. BALB/c nude mice were subcutaneously inoculated with HepG2 cells stably expressing P4HB siRNA or control siRNA. The tumors were harvested after 37 days. Compared with the control group, tumors from the P4HB siRNA group displayed significantly reduced size and weight (Figure 6A–6C). Thus, P4HB promotes HCC tumorigenesis *in vivo*.

## DISCUSSION

In this study, we found that P4HB is upregulated in human HCC. A higher P4HB expression is correlated with more advance disease and poorer patient survival. To our knowledge, this is the first report on P4HB as a potential diagnostic/prognostic marker for HCC. One limitation of the current study is the relatively small sample size (42 HCC patients). Univariate and multivariate analyses are warranted in our future studies to evaluate the potential value of P4HB as an independent prognostic factor for overall and disease-free survival.

In the current study, we also found that P4HB promotes HCC cell growth, migration, and invasion *in vitro* and tumor formation *in vivo*. Intriguingly, P4HB exerts its oncogenic function in HCC at least partially by downregulating GRP78, thereby enhancing EMT of HCC cells. P4HB has been linked to cancer cell resistance to the growth-inhibitory effects of transforming growth factor-β1 (TGF-β1) [16]. In addition, P4HB has been reported to protect cancer cells from drug-induced apoptosis; these anti-apoptotic properties of P4HB in cancer cells have been accredited to its function to ameliorate ER stress through interaction with a variety of protein partners [17, 18]. Our findings revealed that P4HB can promote HCC by enhancing EMT through GRP78 downregulation. Whether this applies to other types of cancer waits to be investigated. Suppression of ER stress may also be a contributing mechanism underlying the oncogenic function of P4HB in HCC. Further experiments are required to answer these remaining questions. Findings from this and further studies may be used to harness development of novel therapeutics for HCC treatment.

## MATERIALS AND METHODS

### Patients and tissue collection

The tumor and adjacent normal tissues were collected from 42 HCC patients who underwent surgery at Shanghai Seventh People's Hospital between 2007 and 2011. The study protocol was approved by the Institutional Research Ethics Committee. All study participants gave written informed consent. Clinicopathological parameters of study subjects are shown in Table 1.

### Cell lines

The HCC cell lines Huh-7, HepG2, PLC5, Hep3B, and SK-Hep-1 and the human normal liver cell line L02 were purchased from the Shanghai Institute of Cell Biology, Chinese Academy of Sciences (Shanghai, China). The cells were cultured in Dulbecco's modified Eagle's medium (DMEM; Invitrogen, USA) supplemented with 10% fetal bovine serum (FBS; Invitrogen), 100 U/ml penicillin (Invitrogen), and 100 μg/ml streptomycin (Invitrogen) at 37°C, 5% CO_2_ in a humidified incubator.

### Quantitative reverse transcription real-time PCR

Total RNA was extracted using the Trizol reagent (Invitrogen) following the manufacturer's instructions. cDNA was synthesized from 1μg RNA using Moloney murine leukemia virus (M-MLV) reverse transcriptase (Invitrogen). The quantitative reverse transcription real-time PCR (qRT-PCR) was carried out using a SYBR Green reaction mix (Applied Biosystems, USA) on an ABI Prism 7900HT sequence detection system (Applied Biosystems). The PCR primer sequences were: P4HB (forward, 5’-GGAATGGAGACACGGCTTC-3’; reverse, 5’-TTCAGCCAGTTCACGATGTC-3’) [12], GRP78 (forward, 5’-GCCTGTATTTCTAGACCTGCC-3’; reverse, 5’-TTCATCTTGCCAGCCAGTTG-3’), and β-actin (forward, 5’-AGCGCGGCTACAGCTTCA-3’; reverse, 5’-GGCCATCTCTTGCTCGAAGT-3’) [9].

### Western blotting

Human tissue samples were homogenized in RIPA lysis buffer (Beyotime, China) and stored at -80°C until analysis. The cells were washed with cold PBS and lysed in lysis buffer (Thermo Scientific, USA) containing a complete protease inhibitor cocktail (Boehringer Mannheim, USA). Tissue or cell lysate samples (20 μg total protein) were separated by SDS-PAGE (10% gel) under reducing conditions and transferred to PVDF membranes (Bio-Rad Laboratories, USA). After blocking in 5% fat-free milk with 0.05% Tween 20 in PBS, the membranes were incubated with anti-P4HB, anti-GRP78, anti-E-cadherin, anti-N-cadherin, anti-vimentin, or anti-β-actin antibody at 4°C overnight. After washing, the membranes were incubated with horseradish peroxidase (HRP)-conjugated secondary antibodies (1:5000) at room temperature for 1 h. Protein bands were visualized using an enhanced chemoluminescence reagent (GE Healthcare, USA). The intensity was measured on a VersaDoc 5000 densitometer (BioRad, USA). All primary and secondary antibodies were purchased from Abcam (Cambridge, United Kingdom).

### Plasmids, siRNAs, and cell transfection

Full-length human P4HB and GRP78 cDNAs were generated by PCR and subcloned into the pcDNA3.1 vector. The siRNAs specific for human P4HB and GRP78 (5’-AAGATGAACTGTAATACGCAA-3’ and 5’-AAGGTTACCCATGCAGTTGTT-3’, respectively) and a scrambled siRNA used as a negative control were designed and synthesized by GenePharma (Shanghai, China). HepG2 or Huh-7 cells were transfected with the pcDNA3.1 vector, pcDNA3.1-P4HB plasmid, pcDNA3.1-GRP78 plasmid, control siRNA, P4HB siRNA, and GRP78 siRNA, in combination or alone using Lipofectamine 2000 (Invitrogen) for 24 h and used in *in vitro* experiments.

### Cell proliferation assay

Cell proliferation was determined using the MTT assay. HepG2 or Huh-7 cells were transfected with plasmids or siRNAs for 24 h. After the transfection, cells were seeded in 24-well plates (2 ×10^4^ cells/well) and cultured for up to 72 h. At specific time points, the medium was removed, and the cells were incubated with MTT solution (0.25 mg/ml) for 1 h. DMSO was added to dissolve the formazan crystals formed, and absorbance at 550 nm was recorded on a spectrophotometer (GE Healthcare, USA).

### Cell migration and matrigel invasion assays

Cell migration was assessed using a Transwell chamber with 8 μm membrane (BD Biosciences, USA). Briefly, 1 × 10^5^ cells were suspended in 500 μl of serum-free DMEM and loaded into the upper chamber. The lower chamber was filled with 1 ml growth medium. After 24 h incubation, the non-migrating cells on the upper surface of the membrane were removed by scraping. The cells that had migrated to the lower surface of the membrane were fixed with 70% methanol, stained with 0.1% crystal violet, and counted under an Olympus IX71microscope. To assess cell invasion, cells were serum-starved overnight and loaded to the upper chamber pre-coated with Matrigel (1 × 10^5^ cells/chamber). After 24 h incubation, the cells that had migrated to the lower surface of the membrane were fixed, stained, and counted as above. Cells in five randomly selected fields of each membrane were counted. Each experiment was conducted in triplicate.

### Immunofluorescence analysis

HepG2 and Huh-7 cells transfected with P4HB siRNA, control siRNA, P4HB, or control vector were grown on 2-well chamber slides (Nalge Nunc International, USA) for 48 h. The cells were subsequently incubated with anti-E-cadherin or anti-vimentin antibody at 4°C overnight. After washing with PBS, the cells were incubated with a FITC-conjugated secondary antibody (Vector Laboratories Inc., USA) at room temperature for 1 h. The cells were then stained with 4’, 6-diamidino-2-phenylindole (DAPI) and examined under a Leica SP2 MP confocal microscope (Leica-Microsystems, Germany).

### Generation of stable cell lines

P4HB siRNA or control siRNA were cloned into a doxycycline-inducible *tet*-on lentiviral vector (Ambion, USA). Constructed lentiviral vectors were generated in HEK293T cells using Trans-Lenti Packaging Kits (Thermo Fisher Scientific Inc., USA). HepG2 cells were infected with viral supernatants (MOI = 2) and selected with 3 μg/ml puromycin (Sigma-Aldrich, USA). Cells stably expressing P4HB siRNA or control siRNA were used for xenograft establishment in nude mice.

### Tumor xenografts in mice

BALB/c nude mice (4–5 weeks of age, 18–20 g) were purchased from the Center of Experimental Animals at Guangzhou University of Chinese Medicine. The animals were and housed in barrier facility on a 12/12-h light/dark cycle. All animal studies were approved by the Ethics Committee of Shanghai Seventh People's Hospital. The mouse xenograft model was established as previously described [19]. In brief, the mice were randomly divided into two groups of five mice each (n = 5 per group). Mice in one group were subcutaneously inoculated with HepG2 cells (5 × 10^6^) stably expressing control siRNA, and those in the other group with HepG2 cells (5 × 10^6^) stably expressing P4HB siRNA. The tumors were harvested on day 37. Tumor volume and tumor weight were determined.

### Statistical analysis

All data are presented as the mean ± SD (standard deviation). Data were interpreted using SPSS 16.0. Multiple comparisons were performed using one-way analysis of variance (ANOVA). Differences with a *P* value less than 0.05 were considered to be statistically significant.
